# Glypican-3 targeted radiopharmaceuticals for hepatocellular carcinoma: a review of molecular platforms

**DOI:** 10.1186/s13550-026-01454-4

**Published:** 2026-05-26

**Authors:** Yue Sun, Lin Qiu

**Affiliations:** 1 Department of Nuclear Medicine, Affiliated Hospital of Southwest Medical University, No. 25, Taiping St, Luzhou, 646000 Sichuan P.R. China; 2 Nuclear Medicine and Theranostics Key Laboratory of Sichuan Province, No. 25, Taiping St, Luzhou, 646000 Sichuan P.R. China; 3 Institute of Nuclear Medicine, Southwest Medical University, No. 25, Taiping St, Luzhou, 646000 Sichuan P.R. China

**Keywords:** Hepatocellular Carcinoma, Glypican-3, Radiopharmaceuticals, Targeted delivery, Theranostics, Pharmacokinetics

## Abstract

**Background:**

Hepatocellular carcinoma (HCC) is characterized by high heterogeneity, making early diagnosis and systemic therapy challenging. Glypican-3 (GPC-3) targeted radiopharmaceuticals are promising tools for HCC. This review summarizes the development of this field from a platform-centric perspective, comparing various molecular targeting vectors designed against GPC-3.

**Main body:**

The development of GPC-3 radiopharmaceuticals has evolved from slow-clearing monoclonal antibodies (mAbs) to rapidly excreted antibody fragments, peptides and other promising platforms. Early platform selection faced a compromise between molecular size and tumor retention. Current strategies tend to optimize pharmacokinetic profiles through chemical modifications, including the application of albumin-binding groups, PEGylation (polyethylene glycol modification), cyclization and integrating these strategies. These engineering approaches aim to provide an optimal signal-to-noise ratio in same-day imaging for diagnosis, while ensuring sustained tumor retention required for targeted radionuclide therapy.

**Conclusion:**

Future clinical translation will require further exploration of optimal pharmacokinetic properties to achieve a better balance between diagnostic and therapeutic capabilities, which is essential to promote GPC-3 theranostics into routine clinical practice. Furthermore, emerging strategies such as shifting towards more potent alpha-radionuclides; moving from single-targeting to multi-targeting strategies, such as GPC-3 and prostate-specific membrane antigen (PSMA); and synergizing radionuclide therapy with immunotherapy (e.g., immune checkpoint inhibitors) are expected to become key to overcoming tumor heterogeneity and prolonging patient survival.

**Supplementary Information:**

The online version contains supplementary material available at 10.1186/s13550-026-01454-4.

## Background

Hepatocellular Carcinoma is a highly heterogeneous malignancy with rising global incidence and shifting etiologies, posing a major health challenge with extremely poor prognosis [1–3].

Current HCC screening relies on ultrasound (often combined with alpha-fetoprotein), which is limited by low sensitivity and leads to an advanced stage diagnosis for many patients [3]. Although central to diagnosis, the sensitivity of Computed Tomography (CT) and Magnetic Resonance Imaging (MRI) is compromised by atypical imaging features, particularly pronounced for small tumors (< 2 cm), making pathological biopsy indispensable [2, 4].

Early efforts in nuclear imaging were also unsuccessful; high glucose-6-phosphatase expression in hepatocytes limited the sensitivity of [^18^F]fluorodeoxyglucose PET. Other traditional metabolic tracers such as choline and acetate also failed to provide a decisive diagnostic advantage [5].

On the therapeutic front, most patients are diagnosed at a late stage, rendering them ineligible for curative treatments. Furthermore, current systemic treatments yield low response rates and are frequently hampered by resistance. Additionally, there are no effective adjuvant therapies to prevent post-operative recurrence [2].

These clinical bottlenecks derive primarily from the profound inter- and intra-tumoral heterogeneity of HCC [6]. This inherent complexity can only be addressed through precision medicine, and Glypican-3 (GPC3) has emerged as a key therapeutic target [2].

Glypican-3 is a cell-surface heparan sulfate proteoglycan overexpressed in 70–80% of HCC tumors. However, it is virtually absent in healthy adult liver tissue and benign hepatic lesions [7], rendering it as an ideal target for both diagnosis and therapy [8]. The diagnostic utility of GPC3 is well-established. It is a diagnostic immunohistochemical marker differentiating HCC from benign lesions, and its soluble form (sGPC3) enables liquid biopsy. Therapeutically, while GPC3 has catalyzed the development of various targeted therapies (e.g., monoclonal antibodies, chimeric antigen receptor T-cell, tumor vaccines, etc.), their clinical efficacy is often hampered by the immunosuppressive tumor microenvironment, systemic toxicities, and the inability to non-invasively monitor whole-body target expression in real-time [9].

In this context, GPC-3 targeted radiopharmaceuticals, particularly those based on the theranostic principle, offer a compelling path forward. Diagnostically, molecular imaging techniques like PET and single-photon emission computed tomography(SPECT) offer a non-invasive, repeatable, and quantitative whole-body assessment of GPC-3 expression. This provides a robust foundation for personalized patient selection and treatment response evaluation. Therapeutically, this approach allows for the targeted delivery of radionuclides to tumor lesions throughout the body, while minimizing systemic toxicity. Moreover, through the crossfire and bystander effects, it can eradicate adjacent tumor cells with low or no GPC-3 expression, offering a powerful tool to overcome tumor heterogeneity [10].

Herein, we systematically review and analyze the recent advancements in GPC-3 targeted radiopharmaceuticals for the whole-body diagnosis and treatment of HCC. We adopt a platform-centric approach to dissect the fundamental trade-offs between these different formats.

## Main text

### The oncogenic role of Glypican-3 in hepatocellular carcinoma

**Fig. 1:**
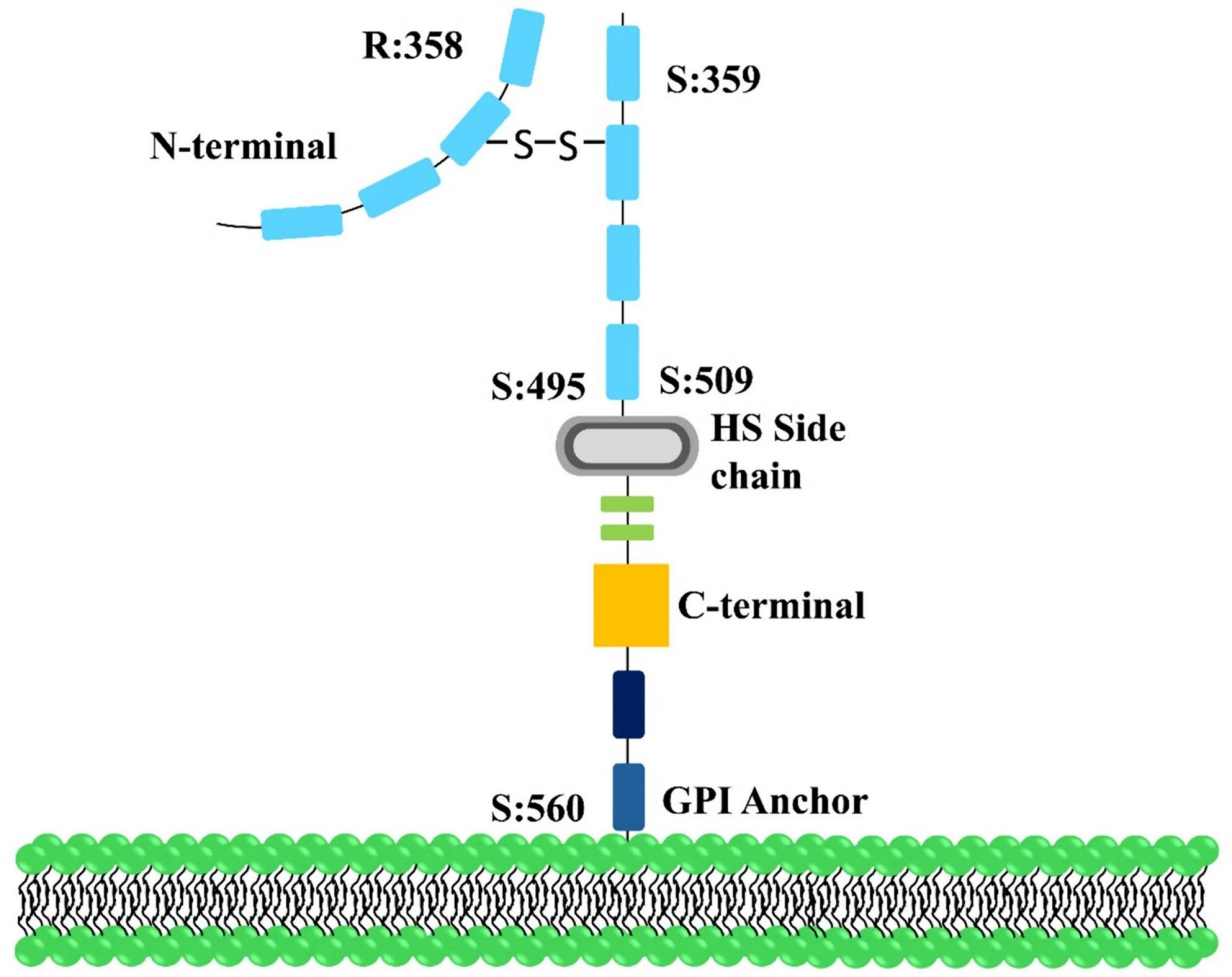
Key structural features of Glypican-3 (GPC-3). The 70-kDa core protein is tethered to the cell surface via a glycosylphosphatidylinositol (GPI) anchor. Following post-translational cleavage by furin at the Arg358-Cys359 site, the resulting subunits remain linked by disulfide bonds. Two HS side chains are attached to the C-terminal subunit. Sheddase cleaves the GPI anchor at Ser560 (Reprinted from Reference [13], licensed under CC BY-NC 4.0.).

GPC3 is a member of the Glypican family of proteoglycan, a group of heavily glycosylated proteoglycans. They are composed of a core protein covalently linked to several linear polysaccharide chains, predominantly heparan sulfate (HS) chains (Fig. 1). Both the core protein and the HS chains play critical roles in oncogenesis and thus serve as therapeutic targets [11].

The GPC3 gene encodes a 70 kDa core protein. The translated polypeptide contains a Furin cleavage site and can be cleaved into two subunits: a 40 kDa N-terminal fragment and a 30 kDa C-terminal fragment. These subunits remain linked by one or more disulfide bonds. The C-terminal portion contains two heparan sulfate (HS) chains and is anchored to the cell membrane by a glycosylphosphatidylinositol (GPI) anchor. This anchor can be cleaved by membrane-bound sheddases, releasing a soluble form of GPC3 (sGPC3) into the circulation [12].

During embryonic development, GPC3 functions as a negative regulator of cell growth and is subsequently silenced in adult tissues. Its re-expression in HCC is driven by the activation of oncogenic pathways involving c-Myc and Yes-associated protein, and the functional loss or silencing of its transcriptional repressor, zinc fingers and homeoboxes 2. In essence, GPC3 is an oncofetal protein whose overexpression correlates with poor prognosis.

GPC3 drives HCC progression through several key mechanisms. The GPC3 core protein stabilizes the binding of Wnt ligands to their Frizzled receptors, while the HS side chains act as a co-receptor to concentrate Wnt ligands at the cell surface, thereby potentiating the canonical Wnt/β-catenin pathway. The HS chains also serve as a co-receptor to capture and present various growth factors like fibroblast growth factor and hepatocyte growth factor [13]. Additionally, GPC3 promotes tumor invasion and metastasis by recruiting M2-polarized tumor-associated macrophages to remodel the tumor microenvironment; it also activates the extracellular signal-regulated kinase pathway to induce epithelial-mesenchymal transition and inhibit apoptosis; and it upregulates hypoxia-inducible factor-1α to drive the Warburg effect [14, 15].

## Molecular platforms for GPC-3 targeting

### Full-size antibodies

Full-size antibodies represent the most established GPC3-targeting vectors, providing high specificity, affinity, and the capacity to generate excellent signal-to-noise ratios. The long biological half-life of antibodies requires radionuclides with compatible physical half-lives, such as ^89^Zr, ^111^In, and ^124^I. For immuno-PET, ^89^Zr (t_1/2_ = 78.4 h) serves as the ideal partner to match the antibody’s circulation time, providing an adequate window for background clearance. Deferoxamine (DFO) is the preeminent chelator for ^89^Zr [16].

**Fig. 2:**
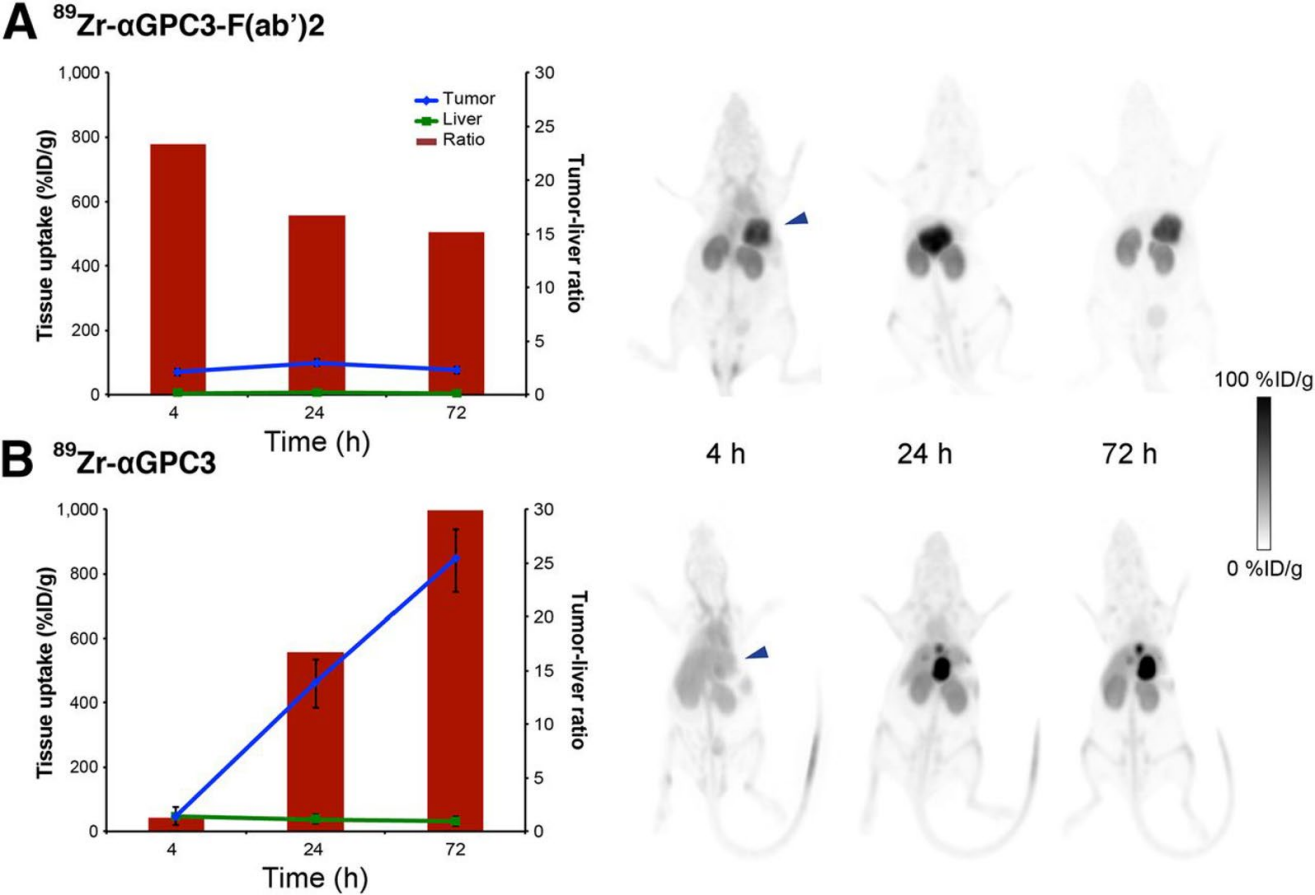
Comparative PET imaging and biodistribution of ^89^Zr-αGPC3-F(ab’)_2_ and full-size ^89^Zr-αGPC3 in HepG2 tumor-bearing mice. (**A**) Representative PET images and corresponding time-activity curves for the ^89^Zr-αGPC3-F(ab’)_2_ fragment. (**B**) Representative PET images and corresponding time-activity curves for the full-size ^89^Zr-αGPC3 antibody. In the quantitative graphs, the solid lines (blue for tumor, green for liver) represent the absolute tissue uptake (%ID/g, left Y-axis), while the bar graphs represent the tumor-to-liver ratio at each time point (right Y-axis). Arrowheads indicate tumor location (This research was originally published in JNM. Sham JG, et al. [30] Glypican-3–Targeting F(ab′)_2_ for ^89^Zr PET of Hepatocellular Carcinoma. J Nucl Med. 2014;55:2032–2037. © SNMMI)

The primary advantage of this platform—exceptional sensitivity—was established early on. In 2014, Sham et al. used ^89^Zr-αGPC3 (a murine mAb) in a cell-line-derived xenograft (CDX) model (Fig. 2B). PET imaging revealed high uptake peaking at > 836.6 ± 86.6% injected dose per gram (ID/g) in 72 h, yielding a peak tumor-to-liver signal ratio of 32.5 and demonstrating sensitivity to resolve sub-millimeter tumors [17]. Subsequently, to define the technology’s detection limits, Labadie et al. systematically evaluated ^89^Zr-αGPC3 PET. The smallest lesion detected was only 330 micrometers in diameter [18]. This indicated that, despite the background noise inherent to slow-clearing agents, the absolute signal intensity of mAbs effectively establishes the detection limit for the whole field.

In parallel, Yang et al. advanced the field by using more clinically relevant patient-derived xenograft (PDX) models established from three different HCC patients with the probe [^89^Zr]Zr-DFO-1G12. PET clearly delineated tumors from healthy liver tissue. Quantitative analysis revealed progressively higher tumor-to-liver (T/L) ratios over time in all models, reaching 4.21 ± 0.64, 2.78 ± 0.26, and 2.31 ± 0.38 in the three PDX models by 168 h [19]. This sustained signal accumulation successfully validated the probe’s efficacy in a predictive, near-clinical setting.

Since the application of murine antibodies is limited by immunogenicity, several groups pursued antibody humanization. Groups led by Natarajan [20] and Dickerson [21] generated the variants [^89^Zr]Zr-Df-H3K3 and [^89^Zr]Zr-DFO-aGPC3H individually via grafting complementarity-determining regions (CDRs) onto human scaffolds. Natarajan’s group confirmed that the humanized agents retained the affinity and specificity of their murine parents in vitro assays, while Dickerson’s group reported statistically indistinguishable tumor-to-liver ratios (12 vs. 11) between the humanized variant and its murine parent in an orthotopic HepG2 xenograft model. This validation effectively decoupled high tumor uptake from immunogenic risk.

While the aforementioned preclinical ^89^Zr-antibody research was ongoing, Carrasquillo et al. conducted the first-in-human GPC3-targeted PET study using [^124^I]I-codrituzumab in 2018 and offered the first direct visualization of intertumoral GPC3 heterogeneity in patients, though it was primarily designed to map biodistribution rather than optimize imaging. In the trial, tumor uptake was highly variable: 6 patients exhibited high uptake (maximum standardized uptake value [SUV_max_] > 9), while seven patients showed relatively less prominent tumor-to-liver ratios, and one patient was negative [22]. This pioneering study emphasized the demand for next-generation GPC3-PET agents with higher sensitivity and greater quantitative accuracy.

Despite their limitations in rapid diagnostics, the structural stability and high tumor retention of full-size antibodies have facilitated considerable progress in radionuclide therapy. In 2019, Ludwig et al. validated the therapeutic efficacy of the β-emitter [^90^Y]Y-DOTA-αGPC3. In an orthotopic HCC model established with HepG2 cells, the agent demonstrated dose-dependent efficacy: after 30 days, the 200 µCi dose group exhibited an increase of 17% while the 300 µCi dose group showed a 35% decrease [23]. This work also demonstrated that ^90^Y-αGPC3 was well-tolerated in this model.

To reduce off-target toxicity, Bell et al. shifted to the more potent, short-range alpha-emitter ^225^Ac. Nonetheless, this increased lethality is accompanied by more significant hematotoxicity across all groups. The side effect may be intensified by long circulatory half-life of full-size antibodies. The authors implemented a dose-optimization strategy to address this safety bottleneck. It revealed that a lower dose (9.25 kBq) conferred a superior survival benefit compared to the higher dose (18.5 kBq) [24]. In a parallel approach, Labadie et al. explored the use of another alpha-emitter to build [^227^Th]Th-αGPC3. Biodistribution analysis confirm the agent’s high target specificity. 23 days after treatment, the 500 kBq/kg dose induced a significant reduction in tumor burden, while the 250 kBq/kg dose also demonstrated clear anti-tumor activity [25]. Although no obvious acute toxicity was observed, the 23-day duration was insufficient to fully assess potential long-term toxicities, especially nephropathy.

Moving beyond standalone agents, Labadie et al. explored the efficacy of the theranostic platform, which provides precision guidance, by pairing ^89^Zr-αGPC3 (imaging) with ^90^Y-αGPC3 (therapy). The efficacy of this sequential theranostic regimen was tested in an orthotopic HCC mouse model. The diagnostic phase first demonstrated high sensitivity: ^89^Zr-αGPC3 enabled the reliable volumetric measurement of small tumors, effectively mapping the targets. Subsequently, compared with the control group, the treatment group showed a significant decrease in serum alpha-fetoprotein levels 30 days after drug administration. Although PET imaging indicated a trend towards a reduction in Gross Tumor Volume (GTV), the authors noted that this difference did not reach statistical significance. Generally, the study successfully validated the feasibility of the GPC-3 theranostic pair [26]. Building upon series of preclinical evidence, Karlsson et al. have recently taken a pivotal step toward clinical translation. They have developed a companion diagnostic tool platform consisting of ^89^Zr-GPC3 and ^225^Ac-GPC3. which is currently undergoing Phase I trials (NCT06345001/NCT06764316) [27]. This also suggests that implementation of a theranostic approach may serve as an indispensable safeguard for precision α-therapy.

### Antibody fragments

Fragments like fragment antigen-binding (Fab), divalent fragment antigen-binding [F(ab’)_2_], and single-chain variable fragment (scFv) remove the fragment crystallizable tail, aiming to engineer a ‘fast-in, fast-out’ kinetic profile. This acceleration enhances tissue penetration and enables rapid blood clearance while retaining sufficient antigen specificity [28]. Their short biological half-life makes them highly compatible with short-lived radionuclides for rapid imaging, including PET isotopes (e.g., ^64^Cu, ^18^F, ^68^Ga) and SPECT isotopes (e.g., ^99m^Tc, ^111^In) [29].

Sham et al. provided the proof-of-concept for this strategy using ^89^Zr-αGPC3-F(ab’)_2_. The F(ab’)_2_ fragment (~ 110 kDa) was generated via enzymatic digestion of the intact parental αGPC3 IgG antibody and showed nearly identical binding affinity. The biodistribution data illustrated the kinetic trade-off: While the fragment successfully accelerated blood clearance (t_1/2_: 11 h vs. 115 h) compared to the full-size parental antibody, its peak tumor uptake plummeted nearly 8-fold (100 ± 21% ID/g vs. 836.6 ± 86.6%ID/g) (Fig. 2A), and the clearance pathway shifted from hepatic to renal, resulting in an attenuated peak liver uptake (5.5 ± 1.1%ID/g vs. 21 ± 5.3%ID/g) coupled with a spike in kidney accumulation (83 ± 12%ID/g vs. 33.4 ± 7.9%ID/g). The rapid clearance of background noise allowed for high-contrast tumor visualization as early as 4 h post-injection, with a tumor-to-liver ratio of 23.2 (vs. 1.2 for the full mAb), which sustained at the 72-hour time point, with a tumor-to-liver ratio of 15.1 (vs. 30.5 for the full mAb). The smallest detectable tumor was 1.5 mm in diameter [30].

The drive for speed pushed the field toward the smaller (30 kDa) single-chain variable fragment (scFv). Guan et al. utilized phage display technology to develop an ^131^I-labeled humanized scFv. As predicted by the size reduction, biodistribution data showed that ^131^I-scFv achieved even faster tumor uptake and more rapid clearance from blood, kidneys, and liver compared to the F(ab’)_2_ fragment [31]. However, the time points selected by the authors were relatively late, leaving the biodistribution and imaging efficacy within the earlier phase (e.g., within 4 h) unexplored.

In addition to the common antibody fragments above, the potential of mid-sized formats was explored by Hanaoka et al., who compared a heavy-chain antibody (HN3, 78 kDa) with a full-size mAb (YP7, 150 kDa) [32]. The study used ^111^In-labeled antibodies for in vivo biodistribution experiments. The quantitative data provide cross-validating evidence that, compared to full-size antibodies, a mid-sized antibody fragment like HN3 also exhibits the key advantages of efficient tumor accumulation, rapid blood clearance, and fast internalization.

The quick washout and renal clearance pathway of fragments demands a short-lived radionuclide. In 2023, Lin et al. developed [^68^Ga]Ga-NOTA-αGPC3-Fab by combining a fully human Fab with ^68^Ga (t_1/2_ = 68 min), thereby significantly diminishing the radiation exposure for patients [33]. PET imaging validated the rapid blood clearance and tumor enrichment. Peak tumor uptake occurred at 2 h post-injection. Benefiting from very low background signals, the probe enabled rapid tumor visualization. At 4 h, tumor-to-blood and tumor-to-liver ratios reached high values of 1.8, and 1.5, respectively. The research also explored a strategy to alleviate Fab nephrotoxicity, demonstrating that pre-injection of sodium maleate diminished renal accumulation by 11.8-fold.

The translational potential of rapid diagnostic kinetics was realized when the same group advanced a novel scFv probe, [^68^Ga]Ga-XH-06, from bench to bedside [34]. This agent pairs scFv with ^68^Ga to push the limits of rapid imaging. In vivo PET imaging obtained clear tumor visualization at 1-hour post-injection, with contrast continuing to improve for up to 4 h in a subcutaneous HCC model. This high-contrast performance translated effectively to orthotopic setting, where biodistribution analysis confirmed a tumor-to-liver ratio of 3.14 at 4 h. First-in-human assessment (7 with HCC, 1 with gastrointestinal stromal tumor) confirmed the safety profile. No adverse events were reported, and high physiological retention was confined to the kidneys. In terms of diagnostic efficacy, the probe detected 100% of HCC lesions, including 5–6 mm nodules, while specificity was validated by a negative gastrointestinal stromal tumor scan. At 2.5 h post-injection, the mean tumor-to-liver ratio in patients was 6.2.

Lin et al. also evaluated the therapeutic potential of these formats by comparing ^177^Lu-labeled scFv, Fab, and full-size mAb for radionuclide therapy (RIT). Preliminary findings indicated that while all agents showed anti-tumor activity, the full-size mAb provided the most potent therapeutic effect and the greatest survival benefit. The study also highlighted nephrotoxicity as a safety bottleneck for small-fragment-based RIT [35]. The data reaffirms that although rapid clearance benefits imaging, therapeutic efficacy depends on retention. In the absence of pharmacokinetic modification, full-size mAbs remain the superior therapeutic framework compared to rapidly excreted fragments.

### Single-domain antibodies

The pursuit of smaller antibody formats has extended beyond Fab and scFv fragments. This led to the development of single-domain antibodies (sdAbs), a category of agents with a molecular weight of merely 12–15 kDa [29]. The unique single-domain structure of sdAbs provides advantages in tissue penetration and rapid clearance for fast imaging applications. Their fast pharmacokinetics are suited for labeling with short-lived radionuclides like ^68^Ga and ^18^F.

In 2022, An et al. reported the first use of a nanobody-based tracer for GPC3 [36]. The group developed a nanobody named G2 (~ 15 kDa) to generate [^68^Ga]Ga-NOTA-G2 and ^18^F-G2 for PET imaging. Both agents clearly delineated GPC3-positive tumors at 1-hour post-injection. While ^18^F-G2 exhibited a better tumor-to-muscle ratio than [^68^Ga]Ga-NOTA-G2 (12.93 ± 3.01 vs. 4.65 ± 1.12), it also showed high physiological hepatic uptake (tumor-to-liver ratio = 0.5), which compromises its utility for diagnosing orthotopic liver tumors. Therefore, [^68^Ga]Ga-NOTA-G2 is a more suitable candidate for HCC imaging. Despite the diagnostic potential of [^68^Ga]Ga-NOTA-G2, its rapid renal clearance carries the risk of nephrotoxicity, hindering its translation into RIT. To address the issue, the researchers fused G2 with an albumin-binding domain (ABD) to create the ABDG2 fusion protein. Biodistribution data revealed that, compared to the parent nanobody G2, [^68^Ga]Ga-NOTA-ABDG2 suppressed renal uptake by nearly 9-fold (1 h SUV_mean_: 0.61 ± 0.11 vs. 5.58 ± 0.93). Although blood retention was initially prolonged, the sustained tumor accumulation along with progressive clearance of background activity led to continuously improving PET image contrast over time.

Optimization can also occur at the atomic level. Fayn et al. evaluated the impact of conjugation strategy by comparing Sortase-mediated site-specific conjugation ([^89^Zr]Zr-ssHN3) with standard random lysine modification ([^89^Zr]Zr-nHN3). HN3 (HN3 VH, 15 kDa) is a human GPC3-specific sdAb. Biodistribution data revealed that the site-specifically conjugated probe (ssHN3) demonstrated superior performance with higher tumor uptake (%IA/g, percentage of injected activity per gram: 7.2 ± 1.2 vs. 5.7 ± 1.8 for nHN3), lower off-target accumulation (particularly in the liver), and a significantly higher tumor-to-liver ratio (3.5 ± 0.5 vs. 1.5 ± 0.5) [37]. However, without pharmacokinetic engineering, renal uptake for both conjugates remained exceptionally high (> 140%IA/g at 1 h), similar to other unmodified sdAbs.

### Polypeptides

Descending further down the size spectrum, peptides (< 10 kDa) offer a more compelling platform for tissue penetration and synthetic versatility. However, this extremely rapid systemic clearance due to miniaturization also leads to insufficient absolute tumor uptake and short tumor retention time, which limits their therapeutic application. This creates a critical engineering task to strike a balance between the rapid clearance required for diagnosis and high tumor retention needed for treatment. Furthermore, some early-developed peptides (L5,TJ12P1) exhibited high hepatobiliary metabolic activity, leading to high background signals in the liver.

The quest for optimal binders generates a diverse chemical library. The L5 peptide [38] has been widely used as a foundational scaffold for molecular imaging probes. Subsequently, phage display technology was used to identify several other peptides, including TJ12P1 [39], TJ12P2 [40], GBP [41] and ALL [42]. Computational design has also led to the development of IPA and 12P [43, 44]. More recently, the structural paradigm has begun to shift from linear chains to cyclic formats such as GPC3-F3 [45] and 10P3Me [46]. This tendency toward macrocyclization culminated in RAYZ-8009 [47], offering a clinical-grade tool for imaging and therapy. The optimization route for the polypeptide is shown in Table 1. The amino acid sequences and molecular designs are shown in Table 2.

**Table 1:** Evolution of pharmacokinetics in GPC-3 targeted peptides

Route	Probe (Ref.)	Engineering Strategy	Affinity (K_d_/IC_50_)	Key In Vivo Metric	Model, Time
**L5**	[^18^F]AlF-NODA-MP-6-Aoc-L5 (Wang 2017)	Baseline (Linear L5 peptide + Aoc spacer)	101 nM (Kd)	T/L = 0.93 ± 0.16	(s.c., HepG2, 1 h)
	[^18^F]AlF-GP2633 (Li 2019)	+ Hydrophilic Linker	63.3 nM (Kd)	T/L = 2.00 ± 0.18	(s.c., HepG2, 1 h)
	[^18^F]AlF-NOTA-IPB-GPC3P (Mo 2024)	+ Albumin Binder (IPB moiety)	High (Cell uptake > GP2633)	Tumor uptake = 4.66 ± 0.22%ID/g T/L = 2.16 ± 0.07 Tumor uptake = 5.22 ± 0.37%ID/g	(s.c., Huh7, 1 h) (Orthotopic, Huh7, 1 h)
**GBP**	[^99m^Tc]Tc-HPG (Xu 2021)	+ Hydrophilic Linker	702.3 nM (IC₅₀)	T/M = 11.55 Tumor uptake = 4.38 ± 0.21%ID/g	(s.c., HepG2, 1 h, Biodist.) (Orthotopic, HepG2, 0.5 h, Biodist.)
	[^68^Ga]Ga-PEG_2_-GBP (Li 2024)	+ Hydrophilic Linker	140 nM (IC₅₀)	Unable to obtain high-contrast images.	(s.c., Huh7)
	[^68^Ga]Ga-ALB-GBP (Li 2024)	+ Albumin Binder	71.5 nM (IC₅₀)	T/L = 1.96 ± 0.11 T/L = 2.29 ± 0.13	(s.c., Huh7, 3 h) (Orthotopic, HepG2, 3 h)
**TJ12P2**	[^18^F]AlF-NOTA-TJ12P2 (Qin 2020)	Baseline	*N/A*	Tumor uptake = 1.82 ± 0.29% ID/g T/L = 2.85 ± 0.64	(Sub-Q, HepG2, 0.5 h)
	[^68^Ga]Ga-T2P [^18^F]AlF-T2P (Chen 2025)	Dual-Targeting (GPC3 + PSMA)	*N/A*	Tumor uptake = 1.75 ± 0.16%ID/g > Monospecific (1.25 or 1.07) T/L = 3.81 ± 0.45	(^68^Ga, s.c., Huh7, 1 h) (^18^F, s.c., Huh7, 1 h, Biodist.)
**Cyclic**	[^68^Ga]Ga-DOTA-F3 (Yan 2021)	Cyclization (Disulfide bond)	~ 50,000 nM (Kd)	T/K ≈ 0.8	(s.c., HepG2, 1 h)
	[^68^Ga]Ga-10P3Me (Wang 2025)	Cyclic + PEG_3_ + ABD (Methylbenzoic acid)	93.8 nM(Kd)	Tumor uptake = 5.62%ID/g T/L = 8.28	(s.c., HepG2, 1 h) (Orthotopic, HepG2-LUC, 1 h)
**Macrocyclic**	[^177^Lu]Lu-RAYZ-8009 (Lin 2024)	Intrinsic Optimization (Macrocyclic)	0.35 nM (Kd, human) 0.42 nM (Kd, mouse)	Tumor uptake = 25.28%ID/g T/L = 108.74 T/K = 1.29	(s.c., HepG2, 1 h, Biodist.)
	[^68^Ga]Ga-RAYZ-8009 (Poot 2024)			T/L > 7.5	(Human, 1 h)

Unless otherwise specified, data represent mean ± SD. Abbreviations: s.c., subcutaneous xenograft; Orthotopic, orthotopic xenograft; Biodist., ex vivo biodistribution data; T/L, tumor-to-liver ratio; T/M, tumor-to-muscle ratio; T/K, tumor-to-kidney ratio; ABD, albumin-binding domain; K_d_, dissociation constant; IC_50_, half-maximal inhibitory concentration

**Table 2:** Summary of sequences and structures of GPC3-targeted peptide-based radiopharmaceuticals

Radiopharmaceutical	Parent Peptide	Targeting Sequence (*N*- to C- terminus)	Spacer / Linker	Chelator	Additional Modifications
**[** ^**18**^ **F]AlF-NODA-MP-6-Aoc-L5**	L5	RLNVGGTYFLTTRQ	6-Aoc	NODA-MP	None
**[** ^**18**^ **F]AlF-GP2633**	L5	GGGRDN-RLNVGGTYFLTTRQ	Peptide spacer (GGGRDN)	NODA-MP	None
**[** ^**18**^ **F]AlF-NOTA-IPB-GPC3P**	L5	GGRDN-RLDVGGTYFLTTRQ*	Peptide spacer (GGRDN) + Branched linker	NOTA	Albumin Binder
**[** ^**99m**^ **Tc]Tc -HPG**	GBP	THVSPNQGGLPS	PEG_4_	HYNIC (with tricine and TPPTS as co-ligands)	None
**[** ^**68**^ **Ga]Ga-PEG**_**2**_ **-GBP**	GBP	THVSPNQGGLPS	PEG_2_	DOTA	None
**[** ^**68**^ **Ga]Ga-ALB-GBP**	GBP	THVSPNQGGLPS	Branched linker	DOTA	Albumin Binder
**[** ^**18**^ **F]AlF-NOTA-TJ12P2**	TJ12P2	SNDRPPNILQKR	None (Direct conjugation)	NOTA	None
**[** ^**68**^ **Ga]Ga-T2P / [** ^**18**^ **F]AlF-T2P**	TJ12P2	SNDRPPNILQKR	Complex linkers	NOTA	Dual-targeting
**[** ^**68**^ **Ga]Ga-DOTA-F3**	F3 (Cyclic)	Sequence not disclosed (7 AAs restricted in 2 cystines)	None (Direct conjugation)	DOTA	None
**[** ^**68**^ **Ga]Ga-10P3Me**	10P3Me (Cyclic)	KCLNHELFQTC	PEG_3_	DOTA	Albumin Binder

*The schematic representation reported in the original text (Mo et al. 2024) appears to lack a glycine residue

Initial exploration focused on L5 peptide. In 2017, Wang et al. synthesized [^18^F]AlF-NODA-MP-6-Aoc-L5 [48]. PET imaging at 1-hour post-injection showed GPC-3-positive tumors clearly, with a tumor-to-muscle ratio of 2.46 ± 0.53, demonstrating good specificity. However, intense background signal in the liver led to a poor tumor-to-liver ratio (0.93 ± 0.16). The hydrophilic Aoc spacer was insufficient to reduce hepatobiliary clearance, prompting further refinement. Subsequently, Li et al. refined the L5 peptide by incorporating a highly polar linkers GGGRDN sequence, producing a novel PET probe [^18^F]AlF-GP2633 [49]. A head-to-head comparison against the parent probe [^18^F]AlF-GP2076 (without the linker) revealed significantly higher tumor uptake (3.37 ± 0.35%ID/g vs. 2.13 ± 0.55%ID/g) and significantly lower liver uptake (1.70 ± 0.26%ID/g vs. 3.70 ± 0.98%ID/g) at 60 min. This modification surged the tumor-to-liver ratio from 0.59 to 2.00, greatly improving image contrast. This confirmed that the strategy successfully shifted the probe’s metabolic pathway from the hepatobiliary to the renal system, but it also raised potential concerns about nephrotoxicity.

In 2020, Qin et al. identified TJ12P2 and constructed the probe [^18^F]AlF-NOTA-TJ12P2 [40]. PET imaging was performed in two subcutaneous HCC models with high GPC-3 expression (HepG2 and SMMC-7721). In static scans 30 min post-injection, tumor uptake reached 1.82 ± 0.29%ID/g and 1.58 ± 0.52%ID/g in HepG2 and SMMC-7721 tumors, respectively, with tumor-to-liver ratios of 2.85 ± 0.64 and 3.21 ± 0.47. This probe showed almost no significant uptake in normal liver, which is a significant improvement over previous TJ12P1 probes (Cy5.5).

In 2021, Xu et al. extended GPC3 peptide research to SPECT imaging [50]. To tackle the high liver uptake issue found in previous PET probes, the team had incorporated a hydrophilic PEG_4_ linker at the N-terminus of the peptide. Consequently, the SPECT probe [^99m^Tc]Tc-HPG was developed based on the GBP peptide. In orthotopic models, SPECT/CT visualized GPC3-positive tumors at all tested time points (0.5, 1, 2, and 3 h) and lesions smaller than 3 mm could be identified. The tumor signal peaked at 1 h. Biodistribution data showed that tumor uptake peaked at 0.5 h (3.45 ± 0.18%ID/g), and the tumor-to-muscle ratio peaked at 1 h (11.55 ± 0.54).

This field has also explored structural rigidification to improve performance. Yan et al. used phage display technology to screen for a cyclic peptide, F3 [45]. This peptide was then coupled with the PET isotope ^68^Ga to obtain the tracer [^68^Ga]Ga-DOTA-F3. It achieved a tumor uptake of 4.50 ± 1.20%ID/g at 1 h—closely matching the kidney signal (5.90 ± 1.70%ID/g). This suggests that cyclization may help mitigate the extreme kidney accumulation commonly observed in linear fragments. However, the dissociation constant (*K*_*d*_) of the F3 peptide with recombinant human GPC-3 protein was 5.02 × 10⁻⁵ M, indicating a very weak binding affinity. This means that other high-performance cyclic peptides need to be screened along this cyclization pathway.

While the aforementioned peptide probes could successfully detect HCC, they were limited by low tumor uptake and short retention, which is unfavorable for targeted radiotherapy. To address this, Mo et al. incorporated an albumin-binding moiety (IPB) to create [^18^F]AlF-NOTA-IPB-GPC3P [51]. In a head-to-head comparison against the predecessor probe [^18^F]AlF-GP2633, tumor uptake surged 6.5-fold at 1 h and 14.4-fold by 2 h (5.05 ± 0.23%ID/g vs. 0.35 ± 0.08%ID/g). Although blood clearance was slower, the dramatic improvement in tumor retention resulted in superior tumor-to-organ ratios. The new probe was also less hydrophilic (distribution coefficient = -1.18 ± 0.06 vs. -2.42 ± 0.09) and had lower kidney uptake. This study clearly demonstrated that introducing an albumin-binding moiety optimized pharmacokinetics.

Concurrently, Li, Mo, and colleagues [52] designed a head-to-head study to directly compare two mainstream optimization strategies: PEGylation vs. albumin-binding. They constructed [^68^Ga]Ga-PEG_2_-GBP and [^68^Ga]Ga-ALB-GBP. In subcutaneous model, PET imaging showed that the PEGylated probe reached peak tumor uptake quickly (2.17 ± 0.13%ID/g at 0.5 h) and was then rapidly cleared. The albumin-binding probe, however, displayed progressive tumor retention (peak: 3.17 ± 0.16%ID/g at 3 h) and successfully delineated intrahepatic boundaries in orthotopic model (T/L ratio: 2.29 ± 0.13 at 3 h). A stark difference was also noted in stability; while only 9.3% of the PEGylated probe survived after 1 h, the albumin-binding analogue retained 96.5% integrity. Ultimately, the data support the albumin-binding moiety as a superior alternative to conventional PEGylation for enhancing the in vivo stability and contrast of GPC3-targeted imaging agents.

To manage tumor heterogeneity, Chen et al. pioneered a dual-specific targeting strategy [53]. They developed a heterodimeric probe, [^68^Ga]Ga-T2P, by conjugating their TJ12P2 with PSMA-617. Head-to-head PET imaging validated its synergy. The dual-targeting agent achieved a tumor uptake of 1.75 ± 0.16%ID/g at 1 h post-injection, outperforming its monospecific counterparts—standalone GPC-3 probe (1.25 ± 0.07%ID/g) and the PSMA probe (1.07 ± 0.06%ID/g). A series of blocking experiments further revealed that GPC-3 plays the dominant role in the binding of this dual-targeted agent.

Early probes based on linear peptides (such as L5, TJ12P1, and TJ12P2) provided initial proof-of-concept for GPC3 targeting, accumulating valuable synthetic and preliminary screening experience for the field. However, as independent validations emerged, the intrinsic specificity of these foundational sequences has been brought into question. Rigorous in vitro evaluations by Berman et al. and Burger et al. [54, 55] utilized isogenic cell lines, scrambled peptide controls, and real-time kinetic analyses to reinvestigate these scaffolds. Strikingly, they reported that at concentrations near their originally reported affinities, the cellular binding patterns of naked L5, TJ12P1, and TJ12P2 were indistinguishable from those of random scrambled sequences. These findings collectively suggest that early phage display screening might have inadvertently enriched target-unrelated peptides (TUPs), or that initial validations yielded false positives due to the absence of robust negative controls. Consequently, while researchers have applied various chemical modifications (e.g., PEGylation, albumin-binders, or dual-targeting motifs) to L5 and TJ12P2 cores to improve pharmacokinetics, the debated specificity of the parent peptides complicates the interpretation of their in vivo success, which may rely on multifaceted systemic dynamics (such as the EPR effect or non-specific retention) rather than pure target-driven affinity.

To overcome the inherent limitations and controversies surrounding conventional linear sequences, the field’s structural paradigm has urgently and necessarily shifted toward highly rigidified, conformationally constrained cyclic platforms. In 2025, a multi-center team led by Wang et al. reported a novel cyclic peptide probe, 10P3Me [46], which integrated PEGylation and an albumin-binding moiety. Microscale thermophoresis analysis showed that the affinity of 10P3Me for GPC3 (*K*_*d*_ = 93.8 nM) was more than three times stronger than the predecessor. 10P3Me clearly delineated intrahepatic lesions and achieved a tumor-to-liver ratio of 8.28 at 1 h. Tumor retention was also significantly prolonged from 30 min for predecessor to 2 h. This work demonstrated that rationally combining multiple optimization strategies can synergistically improve probe affinity, tumor uptake, and imaging contrast.

While academic research explored pharmacokinetic modifiers, the industrial candidate RAYZ-8009 (RayzeBio) took a distinct path focused on molecular intrinsic optimization [47]. Based on a novel macrocyclic peptide, the team engineered a binder with sub-nanomolar affinity (*K*_*d*_ ≈ 0.35 nM) and rapid cellular entry (58.6% internalization at 90 min).This specific combination of high affinity and rapid internalization resolved the classic peptide dilemma—rapid renal clearance sacrifices tumor accumulation. RAYZ-8009 ensures rapid background clearance while maintaining massive tumor accumulation (25.28%ID/g at 1 h), generating a tumor-to-liver ratio of 108.74. Consequently, the agent opened a wide theranostic window. The rapid renal excretion minimized systemic exposure, resulting in a favorable tumor-to-kidney ratio (> 1 from early timepoints, peaking at 22.29). Both ^177^Lu- and ^225^Ac-labeled RAYZ-8009 induced complete and durable tumor regression in animal models, powerfully demonstrating its immense potential as a theranostic agent.

**Fig. 3:**
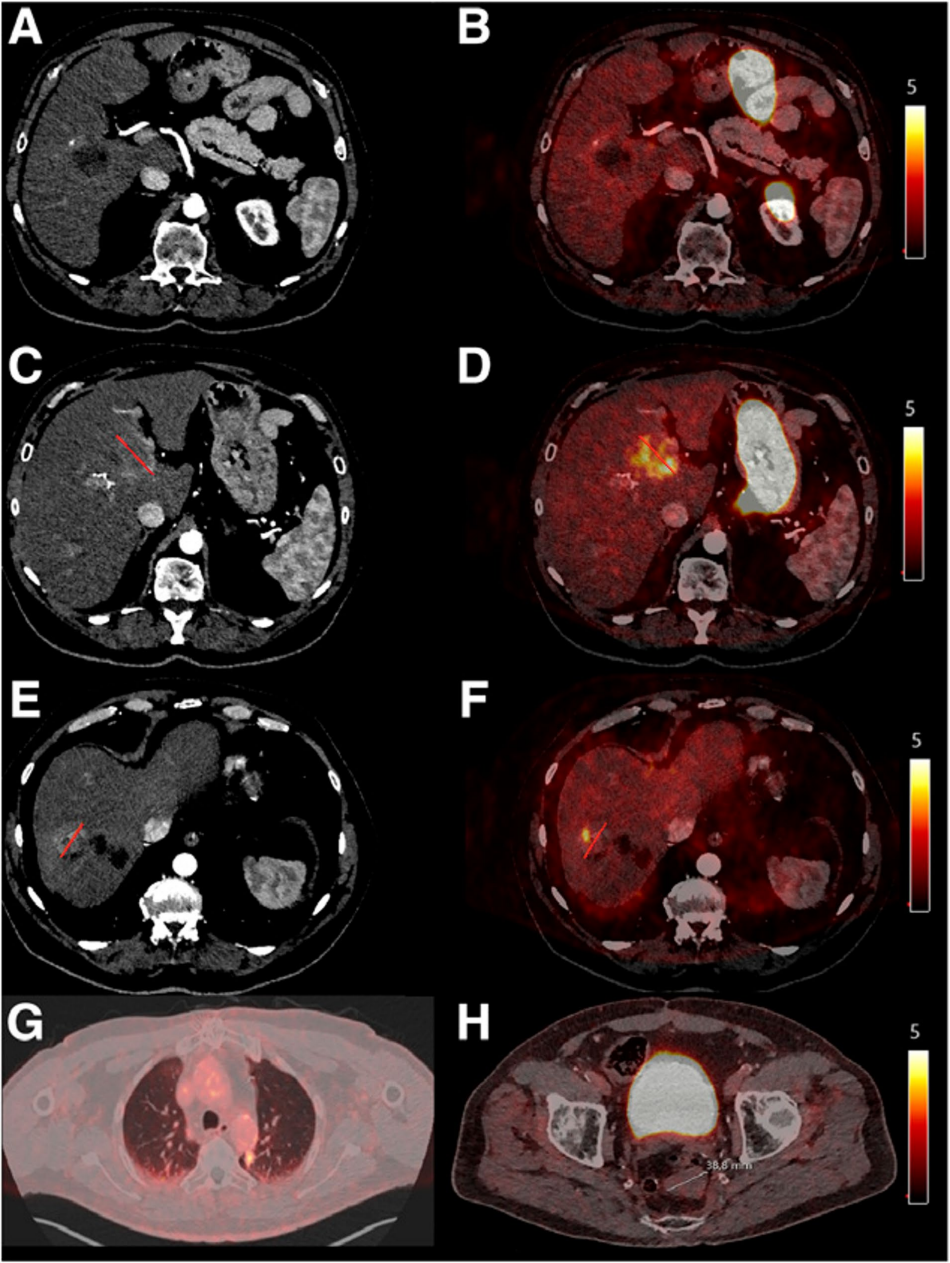
[^68^Ga]Ga-RAYZ-8009 PET/CT imaging in a 66-year-old patient with HCC. The images demonstrate high and specific tracer uptake in GPC-3-positive lesions, including intrahepatic tumors (C, D) and distant metastases (G). Conversely, no significant uptake is observed in biopsy-proven GPC-3-negative lesions, such as an HCC tumor in segment 5/6 (A, B) and a prostate cancer metastasis (H), confirming the tracer’s high specificity in vivo. Red arrows indicate the corresponding lesions and focal accumulations (Arrows have been enhanced for better visibility). (This research was originally published in JNM. Poot AJ, et al. [56] [^68^Ga]Ga-RAYZ-8009: A Glypican-3–Targeted Diagnostic Radiopharmaceutical for Hepatocellular Carcinoma Molecular Imaging—A First-in-Human Case Series. J Nucl Med. 2024;65:1597–1603. © SNMMI.)

Almost immediately, this promising candidate moved to clinical studies. A first-in-human case series by Poot et al. in 24 patients with HCC or hepatoblastoma [56] confirmed the excellent safety profile and a high negative predictive value. PET/CT imaging showed that while background activity washed out rapidly, tumor uptake remained intense (mean SUV_max_ 19.6). This divergent behavior yielded a mean tumor-to-liver ratio exceeding 7.5 within just 60 min. The study also revealed significant inter- and intra-tumoral heterogeneity. The variable uptake of [^68^Ga]Ga-RAYZ-8009 among different lesions in the same patient reflects varying levels of GPC3 expression, thus highlighting the potential of GPC3-targeted PET to guide individualized treatment (Fig. 3). Recently, Viering et al. tested the human dosimetry data to define the operational limits. They calculated that its effective dose is within a clinically safe range and identified the kidneys and bone marrow as the primary dose-limiting organs, dictating the maximum tolerated activities for future radionuclide therapy trials [57].

These remarkable clinical translations powerfully demonstrate that through strict conformational constraint and structural optimization, peptide-based ligands can indeed achieve the high potency and absolute specificity required for GPC3 targeting. Furthermore, this evolution perfectly validates the critical perspectives raised by Berman, Burger, and colleagues: the early, debated linear peptides were not a dead end for this strategy, but rather the essential catalyst that drove the methodology toward maturity and clinical viability.

### Other emerging platforms

Protein scaffolds—engineered, non-antibody proteins (typically 1–20 kDa)—are an emerging platform for GPC-3-targeted radiopharmaceuticals [58]. Protein scaffolds also offer numerous advantages based on their small size and are easily engineered [59]. The first GPC-3-targeting scaffold, an Adnectin-tubulysin antibody-drug conjugate, was reported by Lipovšek et al. Adnectin showed high affinity for GPC-3 (*K*_*d*_ < 30 nM) and kidneys served as the primary clearance organ. This study demonstrated that the Adnectin’s short plasma half-life did not compromise tumor uptake or retention demonstrating a favorable “fast-in, slow-out” kinetic profile [60]. Its findings highlight the scaffold’s potential for GPC-3 radiopharmaceutical applications.

Aptamers—chemically synthesized oligonucleotides—represent another promising class of targeting agents. While their potential has been demonstrated by GPC-3 targeting in non-nuclear imaging [61, 62] and by radiolabeling of other liver cancer aptamers [63], no GPC-3-specific probe has been validated for in vivo SPECT or PET imaging. This research gap may stem from complexities of modifying aptamer pharmacokinetics [64].

## Engineering strategies and clinical translation

To facilitate a comprehensive, platform-centric comparison, the key preclinical and clinical studies discussed in the previous chapter are summarized in Table 3.

**Table 3:** Summary of Key Studies on GPC-3 Targeted Radiopharmaceuticals

Platform	Radiopharmaceutical	Model / Subjects	Significance	Study Phase	Author, date
Full-size Antibodies					
Imaging	[^89^Zr]Zr-αGPC3	Orthotopic CDX, HepG2, Athymic Nu/J mice	Foundational proof-of-concept for GPC-3 immuno-PET.	Preclinical	(Sham et al. 2014)
	[^89^Zr]Zr-DFO-1G12	Orthotopic, HepG2/Hep3B CDX & PDX models, Athymic nude mice	First study achieving high-contrast imaging in clinically relevant PDX models.	Preclinical	(Yang et al. 2014)
	[^89^Zr]Zr-Df-H3K3	Orthotopic PDX, Patient-derived, NSG mice	Validated a humanized GPC3 antibody for PET.	Preclinical	(Natarajan et al. 2021)
	[^89^Zr]Zr-αGPC3	Orthotopic CDX, HepG2-Red-FLuc, Athymic Nu/J mice	Systematically defined the detection limit for micro-lesions (330 μm).	Preclinical	(Labadie et al. 2023)
	[^89^Zr]Zr-DFO-αGPC3H	Orthotopic CDX, HepG2, Athymic nude mice	Confirmed maintained efficacy post-humanization by direct comparison.	Preclinical	(Dickerson et al. 2024)
	^124^I-codrituzumab	14 HCC patients	First-in-human GPC-3 PET study; revealed tumor heterogeneity.	Phase I Clinical	(Carrasquillo et al. 2018)
Therapy & Theranostics	[^90^Y]Y-DOTA-αGPC3	Orthotopic CDX, HepG2, Athymic nude mice	First preclinical validation of GPC-3 targeted RIT, demonstrating dose-dependent tumor inhibition with a β-emitter.	Preclinical	(Ludwig et al. 2019)
	[^225^Ac]Ac-Macropa-GC33	Subcutaneous CDX, HepG2, Athymic nude mice	Explored [^225^Ac]Ac for α-RIT, highlighting significant hematotoxicity and the challenge of balancing efficacy and safety.	Preclinical	(Bell et al. 2020)
	[^89^Zr]Zr/[^90^Y]Y-αGPC3	Orthotopic CDX, HepG2, Athymic Nu/J mice	Validated the feasibility of a sequential [^89^Zr]Zr/90Y GPC-3 theranostic platform.	Preclinical	(Labadie et al. 2021)
	[^227^Th]Th-αGPC3	Orthotopic CDX, HepG2-Red-FLuc, Athymic Nu/J mice	Investigated a potent α-emitter for GPC-3 RIT.	Preclinical	(Labadie et al. 2022)
	[^89^Zr]Zr-GPC3 / [^225^Ac]Ac-GPC3	HCC patients	Pivotal clinical translation of a dedicated [^89^Zr]Zr/[^225^Ac]Ac GPC-3 theranostic pair.	Phase I Clinical (Ongoing)	(Karlsson et al. 2025)
Antibody Fragments					
Imaging	[^89^Zr]Zr-αGPC3-F(ab’)_2_	Orthotopic CDX, HepG2, Athymic Nu/J mice	Pioneering study of a GPC3-targeted fragment, demonstrating faster clearance and enabling earlier imaging than full mAb.	Preclinical	(Sham et al. 2014)
	[^111^In]In-DTPA-HN3	Subcutaneous CDX, A431/G1, Athymic nude mice	Compared a mid-sized HcAb to a full mAb, showing faster clearance with nearly identical tumor accumulation.	Preclinical	(Hanaoka et al. 2015)
	^131^I-scFv	Subcutaneous CDX, HepG2, Nude mice	Developed a humanized scFv for SPECT imaging.	Preclinical	(Guan et al. 2019)
	[^68^Ga]Ga-NOTA-αGPC3-Fab	Subcutaneous CDX, Huh7, Nude mice	First use of a short-lived ^68^Ga-labeled Fab for rapid imaging.	Preclinical	(Lin et al. 2023)
	[^68^Ga]Ga-XH-06 ( [^68^Ga]Ga-NOTA-αGPC3-scFv)	Subcutaneous & Orthotopic CDX, Hep3B, Nude mice 7 HCC patients & 3 healthy volunteers	Clinical breakthrough with a ^68^Ga-scFv, achieving high-contrast, same-day imaging in HCC patients.	FIH Clinical	(Lin et al. 2025)
Therapy & Theranostics	[^177^Lu]Lu-αGPC3-scFv/Fab/mAb	Subcutaneous CDX, Hep3B, BALB/c nude mice	Systematically compared scFv, Fab, and mAb for RIT, concluding full-size mAb is superior for therapy due to longer retention.	Preclinical	(Lin et al. 2025)
SdAb					
Imaging	[^68^Ga]Ga-NOTA-G2 / ^18^F-G2	Subcutaneous CDX, Hep3B, Nude mice	First use of a GPC3-targeted nanobody; ^18^F-labeled version showed high T/M ratio but also high liver uptake.	Preclinical	(An et al. 2022)
	[^68^Ga]Ga-NOTA-ABDG2	Subcutaneous CDX, Hep3B, Nude mice	Introduced an albumin-binding domain (ABD) to a nanobody, prolonging circulation and improving image contrast over time.	Preclinical	(An et al. 2022)
	[^89^Zr]Zr-ssHN3 & [^89^Zr]Zr-nHN3	Subcutaneous CDX, HepG2, Athymic nude mice	Demonstrated that site-specific conjugation (ssHN3) yields superior tumor uptake and T/L ratio compared to random conjugation.	Preclinical	(Fayn et al. 2023)
Polypeptides					
Imaging	[^18^F]AlF-NODA-MP-6-Aoc-L5	Subcutaneous CDX, HepG2, BALB/c nude mice	First study of a GPC3-targeting peptide PET probe, but high liver uptake resulted in a low T/L ratio (0.93).	Preclinical	(Wang et al. 2018)
	[^18^F]AlF-GP2633 [^18^F]AlF-GP2076	Subcutaneous CDX, HepG2, Nude mice	Incorporated a hydrophilic linker to shift clearance from hepatobiliary to renal, successfully improving the T/L ratio to 2.0.	Preclinical	(Y. Li et al. 2020)
	[^68^Ga]Ga-DOTA-F3	Subcutaneous CDX, HepG2, Nude mice		Preclinical	(Yan et al. 2021)
	[^99m^Tc]Tc-HPG	Subcutaneous & Orthotopic CDX, HepG2, BALB/c nude mice	First development of a GPC3-targeted SPECT probe.	Preclinical	(Xu et al. 2021)
	[^18^F]AlF-NOTA-IPB-GPC3P	Subcutaneous & Orthotopic CDX, Huh7, Nude mice	Incorporated an albumin-binding moiety to significantly increase tumor uptake and retention for therapeutic potential.	Preclinical	(Mo et al. 2024)
	[^68^Ga]Ga-ALB-GBP [^68^Ga]Ga-PEG_2_-GBP	Subcutaneous & Orthotopic CDX, Huh7/HepG2-LUC, Nude mice	Directly compared albumin-binding vs. PEGylation, proving albumin-binding is a superior strategy for improving stability and uptake.	Preclinical	(Z. Li et al. 2024)
	[^68^Ga]Ga-T2P	Subcutaneous CDX, Huh7, BALB/c nude mice	Pioneered a dual-specific (GPC3/PSMA) peptide, showing synergistic effects and superior tumor uptake over monospecific probes.	Preclinical	(Chen et al. 2025)
	[^68^Ga]Ga-10P3Me	Subcutaneous & Orthotopic CDX, HepG2, Nude mice	Combined PEGylation and albumin-binding strategies, achieving a high T/L ratio of 8.28 in an orthotopic model.	Preclinical	(Chen et al. 2025)
	[^68^Ga]Ga-RAYZ-8009	24 HCC & Hepatoblastoma patients	Clinical breakthrough; first-in-human study demonstrated excellent safety and high-contrast, same-day imaging.	FIH Clinical	(Poot et al. 2024)
	[^68^Ga]Ga-RAYZ-8009	6 HCC patients	First clinical biodistribution and dosimetry study.	Phase I Clinical	(Viering et al. 2025)
Therapy & Theranostics	[^177^Lu]Lu/[^225^Ac]Ac-RAYZ-8009	Subcutaneous & Orthotopic CDX, HepG2, Nude mice	Demonstrated that both ^177^Lu- and [^225^Ac]Ac-labeled RAYZ-8009 induced complete and durable tumor regression.	Preclinical	(Lin et al. 2024)

### Foundational properties and engineering strategies

The diverse molecular platforms for GPC-3 targeting, ranging from large antibodies to small peptides, are not universally optimal. Instead, each embodies a fundamental trade-off between its physicochemical properties, pharmacokinetics, and ultimate suitability for either diagnostic imaging or radionuclide therapy. These molecular platforms are compared in detail in Table 4.

**Table 4:** Comparative profile of key molecular platforms for GPC3-targeted nuclear medicine

Molecular Platform	Molecular Weight (MW)	Affinity	Tumor Penetration	Clearance Pathway	Biological Stability	Blood Half-Life	Chemical Stability	Immunogenicity Risk	Production Complexity
Full-size Antibody (mAb)	~ 150 kDa	High (pM to nM range)	Poor to moderate	Reticuloendothelial system	High	Long (days to weeks)	Moderate	Moderate to High	High
Antibody Fragments	25–50 kDa (Fab/scFv only)	Moderate to High (nM to sub-nM)	Moderate to Good	Renal	Moderate	Short (hours)	Moderate to High	Low to Moderate	Moderate
Single-domain Antibody (sdAb)	12–15 kDa	High (nM range)	High	Renal	High	Extremely short (minutes to < 1 h)	High	Low to Moderate	Moderate
Peptide	~ 10 kDa	Moderate (µM to nM range)	Very High	Renal	Low (linear, unmodified)	Extremely short (minutes)	High	Low	Low
Protein Scaffold	~ 20 kDa	High (nM range)	High	Renal	High	Short (hours)	High	Low	Moderate
Aptamer	12–30 kDa	High (pM to nM range)	High	Renal	Low (unmodified)	Extremely short (minutes)	Excellent	Very Low	Low

Historically, the development of GPC-3 radiopharmaceuticals was governed by a physical law: the molecular size is the determinant of in vivo behavior. The glomerular filtration threshold (~ 60–70 kDa) determines that smaller molecules are rapidly cleared by the kidneys, resulting in half-lives of probes in the blood ranging from a few minutes for peptides to several weeks for monoclonal antibodies [65, 66]. While smaller size confers superior tumor penetration, the extremely short circulation time often leads to lower absolute tumor uptake and retention compared to mAbs [67]. Compounding this dynamic challenge is the issue of biostability. While full-size antibodies exhibit good stability, smaller molecular platforms like peptides and antibody fragment are metabolically fragile and often require chemical stabilization (such as cyclization or modification) for in vivo applications.

This kinetic dilemma forced a passive selection process: clinicians had to choose between the high contrast of peptides (for imaging) or the high integral dose of antibodies (for therapy) [65]. To alleviate these limitations, academic research has focused on pharmacokinetic modulation, with representative strategies including hydrophilicity engineering and albumin binding. However, the primary goal of these modification strategies still lies in optimizing the diagnostic signal-to-noise ratio of small molecules and avoiding renal toxicity, while full-length antibodies, by virtue of their inherent high retention properties, currently remain the established therapeutic backbone.

Despite this, the advent of RAYZ-8009 marks a unique breakthrough. This macrocyclic peptide demonstrates that small molecules can indeed achieve tumor retention of therapeutic significance. It provides a valuable blueprint for future research, indicating that the therapeutic ceiling of miniaturized drugs is far from reached and is worth further exploration through more advanced molecular designs.

### Translational viability: safety and developability

Safety profiles mirror the kinetic divide established in previous sections. For full-size antibodies, their molecular size and complexity not only confer high immunogenicity, but their prolonged blood half-life also results in hematotoxicity as the primary off-target toxicity, specifically exposing the bone marrow to high radiation accumulation. Conversely, miniaturized platforms trade systemic exposure for renal stress. As they funnel rapidly through the urinary system, the kidneys become the dose-limiting organ.

Clinical translation serves as the ultimate adjudicator, revealing the practical strengths and liabilities of each platform beyond theoretical frameworks. In diagnostic imaging, full-size mAbs pioneered the field but faced kinetic limitations. They validated the target but underscored the logistical burden of using therapeutic antibodies for imaging: sluggish clearance necessitated delayed scanning protocols. Addressing this, the Karlsson group’s recent program represents a maturation of the strategy. By engineering a matched theranostic pair (^89^Zr-GPC3/^225^Ac-GPC3), this approach explicitly manages the safety risks of alpha-therapy through dosimetric guidance. To circumvent antibody limitations, miniaturization strategies have yielded clinical breakthroughs. The synthetic peptide RAYZ-8009 achieved ‘same-day imaging’ in first-in-human studies, delivering high contrast (T/L ratio > 7.5) at 1 h—a feat physically impossible for intact antibodies. Parallel progress is seen in antibody fragments. A ^68^Ga-labeled scFv probe, [^68^Ga]Ga-XH-06, recently demonstrated safety and high-contrast visualization at 2.5 h post-injection in HCC patients, further validating the clinical utility of rapid-clearance kinetics.

Conversely, the therapeutic landscape remains dominated by full-size mAbs, leveraging their prolonged retention for maximal dose delivery. Extensive preclinical data with ^90^Y, ^227^Th, and ^225^Ac have established this platform as the current workhorse for GPC-3 RIT. However, the therapeutic exclusivity of antibodies is being challenged. RAYZ-8009 has demonstrated that optimized peptides can also drive durable tumor regression with ^177^Lu and ^225^Ac, suggesting that small molecules can effectively compete in the therapeutic arena without the pharmacokinetic baggage of large proteins.

The vast differences in developability—manufacturing complexity, cost, and innovation potential—also dictate each platform’s clinical trajectory. As the most mature platform, the full-size mAb has a highly standardized development pathway, but it is constrained by its expensive biomanufacturing model, relying on complex mammalian cell expression systems. Other recombinant protein platforms can often be produced in lower-cost microbial expression systems (e.g., *E. coli*). Peptides and aptamers, produced via an economical ‘chemical synthesis’ model, offer excellent scalability, consistency, and cost-effectiveness.

Beyond cost, the ease of modification determines each platform’s developmental pace. The developability advantage of protein-based platforms (mAbs, fragments, scaffolds) lies in their potential for genetic engineering to optimize biological functions like immunogenicity and affinity. In contrast, the advantage of peptides and aptamers lies in their flexibility for chemical modification, allowing for the systematic optimization of pharmacokinetic deficiencies such as poor stability and short half-life.

### Future perspectives

The immediate priority is to improve the quality of clinical evidence. Current data are primarily limited to small, single-center studies. Future multi-center, randomized controlled trials are essential to validate the diagnostic accuracy and therapeutic survival benefits of leading candidates.

The therapeutic landscape is increasingly moving toward α-emitters over traditional β-sources. While α-particles offer superior therapeutic efficacy, their clinical translation is hindered by safety concerns, notably the off-target toxicity associated with daughter recoil effect. Subsequent research should focus on recoil-resistant chelators and optimized renal-protective dosing strategies to reduce the risk of nephrotoxicity [24].

Finally, to deal with the challenges of tumor heterogeneity, two main strategies are worth considering. First, the development of dual-target or multi-target probes represents a critical direction. Following the pioneering research on GPC-3/PSMA dual-target therapy, future exploration of other synergistic target combinations can achieve more comprehensive targeted therapy for HCC. Second, the combination of GPC-3-targeted drugs with standard treatment regimens, such as immune checkpoint inhibitors, is another promising approach. Radionuclide therapy can convert immunologically “cold” tumors into “hot” tumors, thereby synergistically enhancing the effectiveness of immune checkpoint inhibitors.

## Conclusions

GPC-3-targeted theranostics is rapidly evolving into a clinical reality, driven by the parallel advancement of diverse molecular platforms. By shifting from passive platform selection to active pharmacokinetic engineering, researchers can now decouple molecular size from biological half-life to satisfy distinct clinical objectives. Future progress will require advancing promising candidates through clinical trials while tackling key scientific hurdles, such as tumor heterogeneity and the safety of next-generation radionuclides.

## Supplementary information

Supplementary material 1.

## Abbreviations

ABD: Albumin-binding domain
CDX: Cell line-derived xenograft
CDR: Complementarity-determining region
CT: Computed tomography
DFO: Deferoxamine
Fab: fragment antigen-binding
F(ab')_2_: divalent fragment antigen-binding
GPC3 (GPC-3): Glypican-3
GPI: Glycosylphosphatidylinositol
GTV: Gross tumor volume
HCC: Hepatocellular carcinoma
HIF-1α: Hypoxia-inducible factor-1α
HS: Heparan sulfate
IC_50_: Half-maximal inhibitory concentration
ICI: Immune checkpoint inhibitor
ID/g: Injected dose per gram
IPB: Albumin-binding moiety
K_d_: Dissociation constant
kDa: Kilodalton
mAb: Monoclonal antibody
MRI: Magnetic resonance imaging
PDX: Patient-derived xenograft
PET: Positron emission tomography
PSMA: Prostate-specific membrane antigen
RIT: Radionuclide therapy
scFv: Single-chain variable fragment
sdAb: Single-domain antibody
sGPC3: Soluble form of GPC3
SPECT: Single-photon emission computed tomography
SUV_max_: Maximum standardized uptake value
T/K ratio: Tumor-to-kidney ratio
T/L ratio: Tumor-to-liver ratio
TAMs: Tumor-associated macrophages
