# Supplementary material for: Glypican-3 targeted radiopharmaceuticals for hepatocellular carcinoma: a review of molecular platforms
Source: EJNMMI Res. 2026 May 26;16:112. doi: 10.1186/s13550-026-01454-4 (PMC13402283; doi:10.1186/s13550-026-01454-4)
Supplement: Supplementary file 1 — Supplementary material 1. [file 13550_2026_1454_MOESM1_ESM.docx]

Dear Editor:

Thank you for the opportunity to submit a revised version of our manuscript titled “Glypican-3 targeted radiopharmaceuticals for hepatocellular carcinoma: a review of molecular platforms” to EJNMMI Research.

We greatly appreciate the detailed and constructive guidance from you and the reviewers. We have carefully addressed all the comments. All revisions have been highlighted in the manuscript using Track Changes. Please note that all page and line numbers mentioned in our responses below refer to the revised manuscript with Track Changes.

Below, we provide a point-by-point response to the reviewers’ comments.

**Response to Editor 1’s Comments**

Comment 1:

Page 54. Figure 2. The biodistribution of 89Zr-αGPC3-F(ab')₂ (A) is shown as bar graphs representing the tumor-to-liver ratio at 4, 24, and 72 h. In Figure 2(A), the tumor-to-liver uptake ratio at 24 h (red color) is lower as compared to 4 h.

While in the main paper, the authors state, "89Zr-αGPC3-F(ab′)2 exhibited relatively small changes in tumor uptake over the first 72 h, rising 30 %ID/g between the 4- and 24-h imaging times and then falling 24.3 %ID/g between the 24- and 72-h imaging sessions"

The authors (Sham JG et al.) are encouraged to address this inconsistency with the tumor-to-liver ratio data presented in the figure 2 or revise the statement accordingly.

**Response:**

We thank the reviewer for pointing this out, which allows us to clarify a potential source of confusion regarding the cited data.

Regarding the data in Figure 2A, the figure displays two distinct metrics with separate axes: the absolute tissue uptake (solid lines, left Y-axis, unit: %ID/g) and the tumor-to-liver ratio (red bars, right Y-axis). The original authors' statement about "rising 30 %ID/g" specifically described the absolute tumor uptake (the blue line), which indeed slightly increases from 4 to 24 hours. Conversely, as the reviewer correctly observed, the tumor-to-liver ratio (the red bars) decreases during the same period.

Although the quoted text is not directly present in our manuscript, we realize that broadly citing "(Figure 2)" in our original text might have caused confusion. To eliminate any ambiguity for future readers, we have made the following revisions:

1. In the main text (Page 7, Lines 132 and Page 11, Lines 221), we have changed the citation from "(Figure 2)" to "(Figure 2A)" or "(Figure 2B)" to accurately direct readers to the full-size antibody data.

2. We have updated the Figure 2 legend to explicitly state what the line curves and bar graphs represent. The legend now reads: "In the quantitative graphs, the solid lines (blue for tumor, green for liver) represent the absolute tissue uptake (%ID/g, left Y-axis), while the bar graphs represent the tumor-to-liver ratio at each time point (right Y-axis)."

Comment 2:

Page 56. Figure 3. In the image, the findings were not highlighted with arrows. Authors have copy-pasted the image from the original manuscript where they have mentioned arrows in the legend but not put them in the image. The arrows need to be added to highlight the findings for easier understanding.

**Response:**

We thank the reviewer for this helpful suggestion. The original image from the cited literature did contain thin red arrows; however, compression during the download process reduced their visibility.

To resolve this issue, we have extracted a high-resolution version of the figure from the original PDF source. Furthermore, to ensure the findings are clearly visible to readers for easier understanding, we have digitally thickened and enhanced the original arrows in the updated Figure 3. The figure legend has also been slightly updated to mention this enhancement.

**Response to Editor 2’s Comments**

Comment 1:

All abbreviations used in the text and abstract should be explained the first time they appear.

**Response:**

We thank the reviewer for bringing this to our attention. We have conducted a thorough review of the manuscript and ensured that all abbreviations, including GPC-3, PET, CT, and molecular platform terms (e.g., scFv, Fab), are defined at their first mention in both the abstract and the main text. An updated Abbreviations list has also been provided at the end of the manuscript for clarity.

Comment 2:

Line 136 should be checked and rewritten since "in the three PDX models (HepG2, Hep3B, PC3)" is incorrect. HepG2, Hep3B, and PC3 are xenograft models, not patient-derived.

**Response:**

We thank the reviewer for pointing out this error. We have corrected the text to accurately reflect the original study by Yang et al.

HepG2, Hep3B, and PC3 have been removed from the PDX description, as they are indeed cell line-derived xenograft (CDX) models. We have also clarified that the three PDX models used in their study were established from three different HCC patients, and corrected the time point from 72 h to 168 h to match the original data.

The revised sentence (Page 8, Lines 142-144) now reads: "Quantitative analysis revealed progressively higher tumor-to-liver (T/L) ratios over time in all models, reaching 4.21 ± 0.64, 2.78 ± 0.26, and 2.31 ± 0.38 in the three PDX models by 168 hours".

Comment 3:

The paragraph regarding ^68^Ga-F2/18F-F2 (lines 260-264) is a bit confusing and inconsistent. First, the high liver uptake of ^18^F-G2 is described. Then, an approach to reduce renal uptake is mentioned, followed by results about ^68^Ga-ABDG2. It should be rewritten so that the connections build on one another more effectively.

**Response:**

We appreciate this constructive feedback. We agree that the logical progression between the sections discussing isotope selection and molecular modification needed improvement.

We have carefully restructured and expanded this paragraph (Page 14, Lines 275-281) to clearly separate the two distinct rationales: first, the rationale for isotope selection (comparing ^18^F-G2 and [^68^Ga]Ga-NOTA-G2 to avoid high hepatic uptake), and second, the subsequent molecular modification (introducing the ABD domain to reduce renal uptake and improve pharmacokinetics).

The key revised sentences read: "While ^18^F-G2 exhibited a better tumor-to-muscle ratio than [^68^Ga]Ga-NOTA-G2 (12.93 ± 3.01 vs 4.65 ± 1.12), it also showed high physiological hepatic uptake (tumor-to-liver ratio = 0.5), which compromises its utility for diagnosing orthotopic liver tumors. Therefore, [^68^Ga]Ga-NOTA-G2 is a more suitable candidate for HCC imaging. Despite the diagnostic potential of [^68^Ga]Ga-NOTA-G2, its rapid renal clearance carries the risk of nephrotoxicity, hindering its translation into RIT."

Comment 4:

The chemical structures, or at least the sequences, of GPC3-targeting peptides should be depicted.

**Response:**

We thank the reviewer for this valuable suggestion. We carefully considered drawing the full 2D chemical structures for all the probes. However, we found that several probes contain highly complex structures (e.g., the dual-targeting T2P and the cyclic peptide 10P3Me). More importantly, the exact amino acid sequences or structural formulas for some recent clinical candidates (such as RAYZ-8009 and the F3 peptide) remain proprietary and undisclosed. Therefore, illustrating a unified figure with 2D structures would result in incomplete and potentially misleading representations.

To best address the reviewer's concern and provide clear, accurate information, we have adopted the reviewer's alternative suggestion to depict "at least the sequences." We have introduced a new comprehensive table (Table 2) in the revised manuscript. This table systematically details the parent peptide, the precise amino acid sequence (where publicly available), the spacer/linker types, the chelators, and any additional modifications (e.g., albumin binders, dual-targeting motifs) for each radiopharmaceutical.

Furthermore, during our comprehensive re-evaluation of the original literature to compile this table, we found that we had inadvertently omitted the study on the radiotracer 18F-AlF-NOTA-TJ12P2. We have now added the discussion of this probe to the main text (Page 17, Lines 330-336) and appropriately included its content in Table 1.

Comment 5:

Line 349: "…Wang54…" Please check the reference.

**Response:**

We thank the reviewer for pointing out this oversight. The superscript "54" was a residual formatting error caused by our reference management software (Zotero) during a formatting update. We have removed the stray superscript in the revised manuscript (Page 20, Line 401). The correct citation for this study is properly formatted as [46].

Comment 6:

Line 357-363: The newly published paper of Burger et al. [In Vitro Characterization of the Published Glypican-3-Targeting Peptide TJ12P2 Reveals a Lack of Specificity and Potency. Pharmaceuticals 2025, 18, 1656. https://doi.org/10.3390/ph18111656] should be discussed in this section regarding the binding specificity of the peptide TJ12P2.

**Response:**

We thank the reviewer for pointing out this highly relevant recent publication. We have incorporated the findings of Burger et al. (2025) into the revised manuscript.

To provide a broader context, we also included a similar critical study by Berman et al. (2019), which reported comparable specificity issues for the earlier linear peptides L5 and TJ12P1. Specifically, we noted the lack of in vitro specificity for these linear peptides and hypothesized that their previously reported in vivo tumor uptake may be driven by multifaceted systemic dynamics (e.g., the EPR effect or non-specific retention) rather than purely target-driven affinity.

Furthermore, we used these limitations of linear sequences as a transition to explain why the field is actively shifting toward conformationally constrained macrocyclic platforms (e.g., 10P3Me and RAYZ-8009). These revisions have been highlighted in the revised manuscript (Page 19, Lines 382-401 and Page 22, Lines 441-446).

Comment 7:

Line 457: "A 68Ga-labeled scFv probe recently demonstrated safety and high-contrast visualization…" Which probe are you referring to? Please specify.

**Response:**

We thank the reviewer for pointing this out. The specific probe we were referring to is [68Ga]Ga-XH-06. We have explicitly specified its name in the revised manuscript to avoid any confusion.

The revised text (Page 26, Lines 514) now reads:

"A ^68^Ga-labeled scFv probe, [^68^Ga]Ga-XH-06, recently demonstrated safety and high-contrast visualization at 2.5 hours post-injection in HCC patients…"
